# Recombinant proteasome provides new avenues for anti-malarial drug development

**DOI:** 10.1101/2025.08.13.670186

**Published:** 2025-08-14

**Authors:** Pavla Fajtova, Hanxiao Zhang, Liam Urich, Elany Barbosa da Silva, Cesar Hoffmann da Silva, Jehad Almaliti, Momen Al-Hindy, Evzen Boura, David A. Fidock, Laura A. Kirkman, Gang Lin, Matthew Bogyo, William H. Gerwick, Jianhua Zhao, Anthony J O’Donoghue

**Affiliations:** 1 Center for Discovery and Innovation in Parasitic Diseases, Skaggs School of Pharmacy and Pharmaceutical Sciences, University of California San Diego, La Jolla, CA, USA.; 2 Cancer Metabolism and Microenvironment Program, Sanford Burnham Prebys Medical Discovery Institute, La Jolla, 92037, USA; 3 Pharmaceutical Synthesis Group (PHARSG), Universidade Federal do Rio Grande do Sul, Porto Alegre, RS, Brazil; 4 Department Pharmaceutical Sciences, College of Pharmacy, The University of Jordan, Amman, Jordan.; 5 Scripps Institution of Oceanography, University of California San Diego, La Jolla, CA, USA.; 6 Institute of Organic Chemistry and Biochemistry AS CR, v.v.i., Prague, Czech Republic.; 7 Department of Microbiology and Immunology, Columbia University Medical Center, New York, New York 10032, United States.; 8 Department of Microbiology and Immunology, Weill Cornell Medicine, New York, New York 10065, United States.; 9 Department of Pathology, Stanford University School of Medicine, Stanford, California 94305, United States.

## Abstract

The *Plasmodium falciparum* 20S proteasome (Pf20S) is a promising antimalarial target. Therapeutic development has previously relied on native purifications of Pf20S, which is challenging and has limited the scope of previous efforts. Here, we report an effective recombinant Pf20S platform to facilitate drug discovery. Complex assembly is carried out in insect cells by co-expressing all fourteen subunits along with the essential Pf chaperone homolog, Ump1. Unexpectedly, the isolated proteins consisted of both a mature and an immature complex. Cryo-EM analysis of the immature complexes revealed structural insights detailing how Ump1 and β2/β5 pro-peptides coordinate β-ring assembly, which differ from human and yeast homologs. Biochemical validation confirmed that β1, β2, and β5 subunits of the mature proteasome were catalytically active. Clinical proteasome inhibitors, bortezomib, carfilzomib and marizomib were potent but lacked Pf20S selectivity. However, the tripeptide-epoxyketone J-80 potently and selectively inhibited Pf20S β5 (IC_50_= 22.4 (1.3) nM, 90-fold over human β5), with cryo-EM elucidating the structural basis for its specific binding. Further evaluation of novel Pf20S-selective inhibitors such as the reversible TDI-8304 and irreversible analogs 8304-vinyl sulfone and 8304-epoxyketone confirmed their potency and selectivity over the human constitutive proteasome. This recombinant Pf20S platform facilitates detailed biochemical and structural studies, accelerating the development of selective antimalarial therapeutics.

## Introduction

The ubiquitin-proteasome system is vital for protein degradation and maintaining cellular balance in eukaryotes, making it an appealing target for new therapies [1, 2]. In human medicine, proteasome inhibitors have achieved remarkable clinical success, particularly in treating multiple myeloma. Drugs like bortezomib, carfilzomib, and ixazomib have transformed patient care [3–6]. This success has spurred research into using proteasome inhibition as a strategy to combat parasitic infections. Recent studies have identified significant structural and functional differences between human and parasitic proteasomes, creating opportunities for developing selective inhibitors [7–10]. Notable progress has been made in targeting the proteasomes of protozoan parasites such as *Leishmania donovani* and *Trypanosoma cruzi*. Pharmaceutical companies like GSK and Novartis are developing parasite-specific proteasome inhibitors with minimal cross-reactivity with the human enzyme [11, 12].

*Plasmodium falciparum* (Pf), the causative agent of the most lethal form of malaria, relies heavily on its proteasome for development and survival throughout its complex life cycle [13, 14]. The Pf 20S proteasome (Pf20S) has been recognized as a promising drug target, with pioneering work by multiple research groups establishing its potential. It has been demonstrated that the parasite’s proteasome activity is critical during the intraerythrocytic stages and selective inhibitors with antimalarial activity have been identified [7, 15–17]. In addition, natural products and their derivatives have been discovered that inactivate the Pf20S [18, 19]. Importantly, proteasome inhibitors can overcome resistance to frontline antimalarials [20]. While most inhibitors directly interact with the substrate binding pocket and catalytic threonine, others have been discovered that bind at adjacent sites [7, 21]. Collectively, these studies underscore the importance of Pf20S as a therapeutic target against malaria.

Despite the promising nature of Pf20S as a drug target, biochemical and structural studies have been hindered by the difficulty in obtaining sufficient quantities of the highly pure native enzyme. Recombinant expression systems using insect cells have successfully overcome similar challenges for human, *Trichomonas vaginalis*, and *Trypanosoma cruzi* proteasomes [22–24]. These systems have produced fully functional proteasomes, facilitating structural and functional studies that advance drug discovery. However, the recombinant expression of a functional Pf20S has remained elusive.

In this study, we successfully generated a recombinant Pf20S proteasome by co-expressing all fourteen constituent subunits (seven α and seven β) along with the Ump1 chaperone in an insect cell expression system. The Ump1 chaperone proved crucial for proper assembly, as no functional enzyme complex was detected without co-expression of the chaperone, consistent with findings in other organisms [23, 25, 26]. The recombinant proteasome exhibits biochemical and structural properties identical to those of the native enzyme isolated from parasite cultures. This recombinant enzyme enabled the discovery of a β5-selective substrate and allowed for extensive inhibition studies with a diverse panel of inhibitors. Furthermore, structural studies revealed how a tripeptide-epoxyketone inhibitor binds with high affinity to the β5 subunit. Overall, the recombinant expression system overcomes the limitations associated with native proteasome isolation and establishes a platform for detailed investigation into the structure and function of protozoan proteasomes [27, 28]. This advancement should accelerate the development of novel antimalarial therapeutics with reduced host toxicity.

## Results:

### Assembly and isolation of recombinant Pf20S

The *P. falciparum* proteasome is a validated target for antiparasitic drug development. However, a key bottleneck exists in isolating Pf20S from parasite extracts. The published purification protocols involve two to five time-consuming chromatography steps, yielding limited protein quantities that are only sufficient for small scale biochemical or structural studies ([7, 9, 17, 21] This necessitates regular isolation of fresh proteasome, which in turn requires continuous culturing of the parasite. For other anti-microbial drug targets, the availability of recombinant proteases has facilitated high-throughput screening, concentration-response studies or structural studies [29–31]. Therefore, we set out to establish a recombinant platform for the Pf20S. Encouragingly, our recent work demonstrated that the *Trichomonas vaginalis* proteasome (Tv20S) can be successfully expressed in an Sf9 insect cell system, paving the way to consider additional parasite proteasome targets.

**Figure 1.**
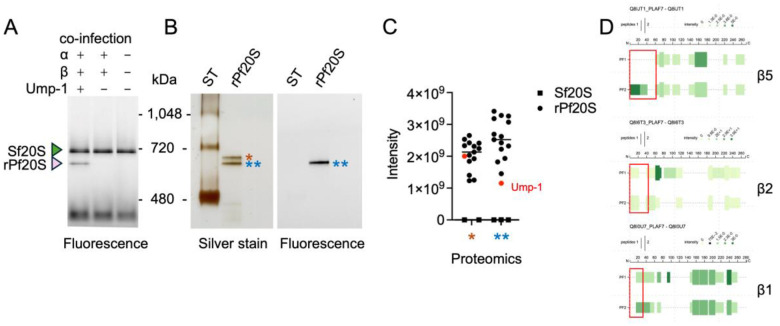
Characterization of recombinant Pf20S proteasome. **A.** Native-PAGE analysis of insect cell lysates following baculovirus co-infection, labeled with Me4BodipyFL-Ahx3Leu3VS fluorescent activity-based probe. **B.** Native-PAGE gel of purified recombinant Pf20S proteasome. Gels were visualized by silver staining (total protein) and fluorescent scanning (active proteasome complexes). **C.** Relative proteomics intensities of proteasome subunits identified from gel bands excised from panel B. **D.** Peptigram showing sequence coverage of identified proteasome peptides from mass spectrometry analysis. Source data are provided in the Source Data file.

The genome of *P. falciparum* encodes seven α and seven β subunits of Pf20S, all of which align with homologous subunits in the human constitutive proteasome (**Fig. S1**). The challenge in generating recombinant Pf20S proteasomes lies in the assembly of the different subunits, which need to be precisely arranged in the correct order. Initial attempts of expression and purification showed that only the proteasome from the host insect cells, *Spodoptera frugiperda* 20S proteasome (Sf20S) was detectable by native PAGE when imaged using the activity-based probe, Me4BodipyFL-Ahx3Leu3VS (Fig. 1A). These studies indicated that assembly and maturation of recombinant Pf20S was unable to occur using the host chaperone proteins. We and others have previously highlighted the importance of a chaperone protein called Ump1 for the expression of a fully functional proteasome [23, 25, 26]. Using protein alignment searches, we identified a homolog of Ump1 in the *P. falciparum* genome (Uniprot ID: A0A5K1K941). Co-expression of this gene with the α and β subunits resulted in the successful isolation of the recombinant 20S proteasome on the native gel using the activity-based probe and could be clearly differentiated from the host proteasome by its different molecular weight and or charge. The recombinant protein complex contains a C-terminal twin-strep tag on the β7 subunit and can be selectively enriched from the cell lysates using a streptavidin column (Fig 1A).

Unexpectedly, analysis of the silver-stained native gel after streptavidin enrichment revealed two protein complexes of similar size (Fig 1B). Both proteins were distinct in molecular weight and charge from the insect proteasome. However, upon addition of the activity-based probe only the lower band was labelled. These findings suggest that the lower band represents the mature, functional Pf20S proteasome, whereas the upper band likely corresponds to an immature proteasome complex. We attempted to separate these complexes by size exclusion chromatography but they eluted as a single peak.

We hypothesized that the difference between the upper and lower band could be attributed to the retention of propeptides from the β1, β2, and β5 subunits and the association of Ump1 chaperones which would change the overall molecular weight and charge. To test this, the upper and lower bands were excised from the silver-stained gel (Fig. 1B) and subjected to proteomics analysis by searching against the *S. frugiperda* and *P. falciparum* proteome. In each band, the seven α and seven β subunits of Pf20S were amongst the most abundant proteins present (Fig. 1C). Ump1 was lower in abundance in the lower band indicating that it may have been degraded by the matured 20S. Several insect proteasome subunits were also found but their peak intensities were more than 1000-fold lower in abundance than the subunits of *P. falciparum* (**File S1**), therefore ruling out the presence of appreciable levels of chimeric insect proteasomes. We next looked at the peptides derived from the propeptide region of the catalytic subunits to determine if they were higher in abundance in the upper band relative to the lower band. For all three catalytic subunits, the propeptide sequences were 1.45-fold to 6.9-fold more abundant in the upper band while the average intensity for peptides derived from the mature region of the subunits showed no difference (Fig. 1D). These studies strongly indicate that the upper band consists of an immature proteasome complex. When considering both the upper and lower bands together, a yield of 1 mg per liter at >98% purity was achieved for recombinant Pf20S using the insect expression system.

### Structural evaluation of immature 20S proteasomes

**Figure 2.**
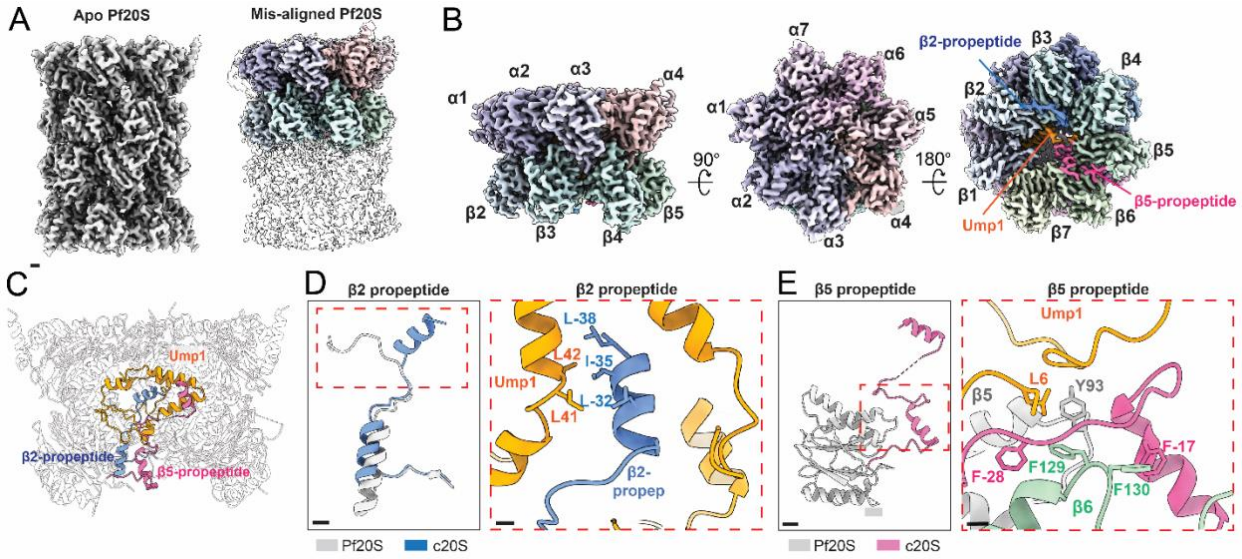
Incorrectly assembled half-Pf20S displays distinct β2 and β5 pro-peptides compared to the human 20S. **A**. Cryo-EM map of incorrectly assembled half-Pf20S, showing top-down view and bottom-up view. **B**. Atomic model of the half Pf20S, illustrating Ump1 and β2 and β5 pro-peptides positioned within the antechamber formed by the α-ring and β-ring. **C**. Interaction between Pf20S Ump1 and N-terminal helix of β2 pro-peptide. **D**. Overlay of β2 pro-peptides from human 20S (light grey) and half Pf20S (blue). Red box highlights the N-termini of the two β2 pro-peptides, which adopt different conformations. **E**. Atomic model of Pf20S β5 pro-peptide (hot pink) and the rest of β5 (light grey). The N-terminal region of β5 pro-peptide forms an α helix (hot pink) and interacts with β6 subunit (light green) and β5 subunit (light grey).

To gain structural insights into both the mature and immature proteasomes, we analyzed the recombinant Pf20S using single particle cryogenic electron microscopy (cryo-EM). Two-dimensional classification of the cryo-EM images revealed the presence of both correctly and incorrectly assembled complexes (**Fig. S2**). Normal assembly involves the association of the β-rings from two half proteasomes to form the nascent core particle, which is then remodeled by the successive removal of the amino-terminal propeptides of β1, β2, β5, β6 and β7 followed by Ump1 degradation. The incorrectly assembled Pf20S complexes consist of two half-Pf20S sub-complexes that are misaligned at the β-β dimerization interface. Three-dimensional refinement of the misaligned complexes resulted in a cryo-EM map where one half-Pf20S displayed high-quality density at ~3.0 Å resolution, while the other half-Pf20S showed low-quality cryo-EM density (Fig. 2A). This suggests that the two half-Pf20S sub-complexes are misaligned in multiple configurations, resulting in blurring of the cryo-EM density after averaging. Analysis of the high-resolution half-Pf20S revealed clear density for Ump1, in addition to the pro-peptides of β2 and β5 within the antechamber. However, no density for the β1 pro-peptide was observed in the cryo-EM map (Fig. 2B).

The structure of the half-Pf20S reveals how the β2 and β5 pro-peptides cooperate with Ump1 to coordinate β-ring assembly. Ump1 adopts a conformation similar to the human homolog POMP. Meanwhile, the *Plasmodium* β2 and β5 propeptides show structural differences compared to their mammalian and yeast counterparts. In the human proteasome, the C-terminus of the β2 propeptide forms a loop that points toward the wall of the antechamber [32, 33], while the C-terminus of the β2 pro-peptide of Pf20S adopts an α-helical conformation and points toward the center of the antechamber (Fig. 2C). This α-helix is stabilized by hydrophobic interactions involving Leu41 and Leu42 of Ump1 and Leu(−32), Ile(−35), and Leu(−38) of the β2 propeptide (numbering corresponding to the mature sequence) (Fig. 2D). Our structure also provides a nearly complete model of the β5 pro-peptide, shedding light on how it facilitates β-ring assembly. Starting from the catalytic threonine (Thr1), the β5 pro-peptide adopts a loop-helix-loop-helix conformation that is similar to the β2 pro-peptide (Fig. 2E). The N-terminal α-helix is stabilized by π-stacking between Phe(−17) of β5 and Phe130 of β6 (Fig. 2F). Additionally, the loop between the two α-helices in the β5 pro-peptide is sandwiched between Ump1 and β6 and is stabilized by stacking of β5 Phe(−28), β6 Phe129, and β5 Tyr93 to form a hydrophobic pocket that is occupied by Ump1 Leu6 (Fig. 2F). These results reveal how the β5 pro-peptide coordinates with Ump1 to recruit β6 during β-ring assembly.

Comparison of the recombinant Pf20S structures with native Pf20S (PDB: 7LXU) offers insights into the factors contributing to the incorrect assembly of the higher molecular weight complex. In the native enzyme, the C-terminal tails of β1 and β7 subunits coordinate the dimerization of two half-proteasomes by extending across the dimerization interface to bind the opposing half-20S complex [32, 33]. However, for the recombinant Pf20S the C-terminus of β1 is unbound in the structure (**Fig. S3**) and the absence of this dimer-stabilizing interaction likely reduces the affinity of half-Pf20S to each other. This reduced affinity likely contributes to the incorrect assembly observed in approximately 50% of the total Pf20S population. Fortunately, these incorrectly assembled Pf20S complexes will not affect our downstream biochemical studies as they are not catalytically active due to their inability to be labelled by the activity-based probe. Consequently, we adjusted the active enzyme concentration to represent 63% of the total protein concentration for subsequent studies, which were performed extensively to validate this recombinant enzyme as a suitable substitute for the native form.

### Biochemical validation of recombinant Pf20S

**Figure 3.**
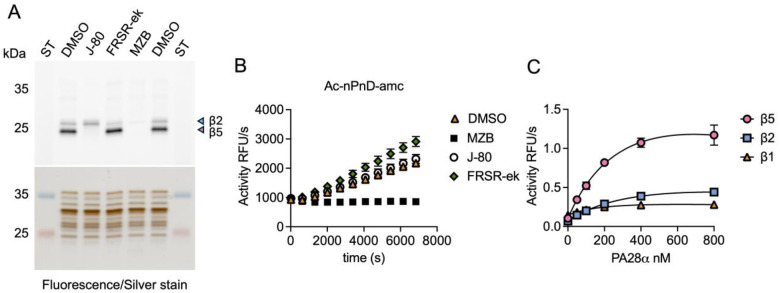
Biochemical characterization of proteasome activity and regulation. **A.** SDS-PAGE analysis of proteasome samples. Upper panel: fluorescence detection using Me4BodipyFL-Ahx3Leu3VS activity-based probe showing protein bands at molecular weights corresponding to β2 and β5 proteasome subunits. Lower panel: silver staining. **B.** Time-course analysis of proteasome caspase-like (β1) activity using Ac-nPnD-amc fluorogenic substrate. Activity measured in the presence of proteasome inhibitors. Data shown as relative fluorescence units (RFUs) over time. **C.** Concentration-response curves showing the effect of PA28α regulatory subunit on proteasome activity. Different proteasome subunits (β5, β2, β1) show distinct responses to increasing PA28α concentrations (0–800 nM).

To assess subunit activity, the purified recombinant enzyme was incubated with the activity-based probe, and the labeled subunits were visualized on a denaturing protein gel. Previous studies had established that this probe selectively labels only the β2 and β5 subunits of the native enzyme, therefore, only two bands were anticipated for the recombinant enzyme. Indeed, two distinct bands appeared at 25 and 23 kDa, confirming the functionality of the β2 and β5 subunits (Fig. 3A). Our previous studies revealed that the tripeptide epoxyketone inhibitor, J-80, is a potent and specific inhibitor of the native Pf20S β5 subunit [34]. Preincubation of the recombinant enzyme with J-80 prior to labeling active subunits with the probe resulted in the elimination of the lower band. This confirmed that the lower band was indeed the β5 subunit. In a parallel experiment, the enzyme was preincubated with Ac-Phe-Arg-Ser-Arg-epoxyketone (FRSR-ek), a tetrapeptide inhibitor developed by our group to inhibit the β2 subunit of *T. vaginalis* [35]. This inhibitor was predicted to bind the Pf20S β2 subunit due to its trypsin-like specificity. Indeed, FRSR-ek decreased the labeling of the upper β2 band with the probe. As a control, the broad-spectrum inhibitor marizomib (MZB) eliminated the labeling of both bands. To confirm β1 subunit activity, recombinant Pf20S was assayed using a previously established fluorogenic substrate: the acetylated tetrapeptide Ac-Nle-Pro-Nle-Asp linked to a C-terminal 7-amino-4-methylcoumarin fluorescent reporter group (Ac-nPnD-amc). Pf20S-mediated cleavage of this substrate was inhibited by the broad-spectrum inhibitor MZB (which targets all subunits), but not by the β5 inhibitor J-80 or the β2 inhibitor FRSR-ek (Fig. 3B). In summary, a combination of the gel-based assays and the fluorescent plate assay confirmed that the β1, β2, and β5 subunits are functionally active in the recombinant Pf20S enzyme.

To ensure a complete set of subunit-specific substrates was available for microwell plate assays with the recombinant Pf20S enzyme, we evaluated the reporter substrates Z-VLR-amc and Suc-LLVY-amc for their cleavage by the β2 and β5 subunits, respectively. Although both substrates were efficiently cleaved, a key difference emerged: activity with Z-VLR-amc (the β2 substrate) was effectively inhibited by FRSR-ek (a β2 inhibitor), whereas Suc-LLVY-amc (the putative β5 substrate) was not completely inhibited by the β5 inhibitor, J-80. This finding indicates that Suc-LLVY-amc may be cleaved by more than one proteasome subunit. Consequently, we evaluated a new β5 substrate, Ac-Phe-Asn-Lys-Leu-amc (FNKL-amc), which was recently developed for assaying the *Schistosoma mansoni* 20S proteasome [36]. We found that Pf20S efficiently cleaved FNKL-amc. Importantly, this substrate proved specific for the β5 subunit, as its cleavage was completely inhibited by J-80 (**Fig. S4**).

Previous studies have shown that human PA28α activates the native Pf20S [8]. To assess the effect of PA28α on the activity of each catalytic subunit in recombinant Pf20S, fluorogenic peptide substrates specific for β1, β2, and β5 were employed: Ac-nPnD-AMC (β1), Z-VLR-AMC (β2), and Ac-FNKL-AMC (β5). With fixed concentrations of enzyme and substrate, assays were performed in the presence of increasing PA28α concentrations up to 800 nM. Subunit activity increased linearly as PA28α concentration increased from 0 to 200 nM, with a less pronounced increase between 200 and 800 nM (Fig. 3C). PA28α is known to bind to the α-rings of the proteasome, which enhances substrate access to the catalytic core. At a concentration of 200 nM PA28α, the activity of the β5 subunit increased 8-fold, while β1 and β2 subunit activities increased 4-fold and 2-fold, respectively. Taken together these studies indicate that the optimal ratio of recombinant Pf20S to PA28α is 1:200. Consequently, we used PA28α at this optimal ratio for all downstream enzyme assays.

### Assessment of clinical proteasome inhibitors as potential anti-malarial inhibitors

**Figure 4.**
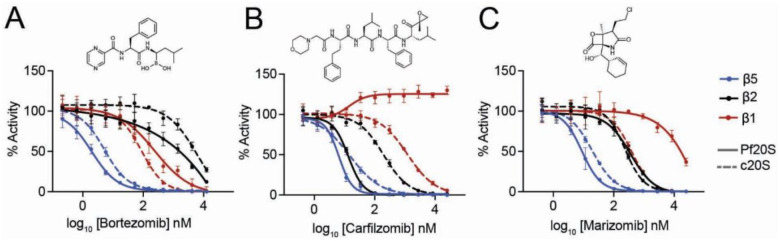
Testing clinical proteasome inhibitors. Chemical structures and concentration response assays for **A**. bortezomib, **B**. carfilzomib and **C**. marizomib against Pf20S (solid lines) and human c20S (dashed lines). Assays were performed in technical triplicate reactions using β5, β2 and β1 specific substrates.

Given the clinical success of proteasome inhibitors like carfilzomib and bortezomib in treating multiple myeloma, and the ongoing clinical trials of the brain-penetrable inhibitor marizomib for glioblastoma, we aimed to directly compare the potency of these compounds against both Pf20S and human constitutive 20S (c20S) proteasomes. Although these inhibitors are known to be toxic to human cells, our goal was to determine if any could serve as a viable starting point for a dedicated anti-malarial drug development program. Bortezomib primarily targeted the β5 subunits of both c20S (IC_50_ = 5.2 ± 0.5 nM) and Pf20S (IC_50_ = 1.9 ± 0.3 nM), and it also inhibited the β1 and β2 subunits of both enzymes, albeit at increasingly higher concentrations (Fig 4A). Carfilzomib inhibited both the β5 (IC_50_ = 6.5 ± 0.4 nM) and β2 (IC_50_ = 13.4 ± 0.8 nM) subunits of Pf20S, but it also targeted the c20S β5 subunit (IC_50_ = 15.2 ± 1.9 nM) within a similar concentration range (Fig. 4B) Finally, marizomib inhibited the β5 subunits of both Pf20S and c20S with IC_50_ values in the 8–20 nM range, while also targeting both β2 subunits (Pf20S and c20S) and the c20S β1 subunit with IC_50_ values of ~300 nM (Fig. 4C). Collectively, these findings demonstrate that the clinical proteasome inhibitors tested exhibit no significant selectivity for Pf20S over c20S, which therefore makes them unsuitable as direct anti-malarial therapies. Consequently, they were not deemed useful starting points for specific anti-malarial drug development efforts.

### J-80 inhibition of Pf20S β5 revealed by structural studies

**Figure 5.**
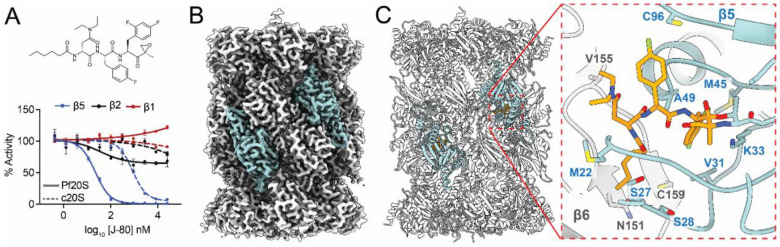
JAM-1–80 covalently binds to Pf20S β5 binding pocket. **A)** Structure of J-80 and concentration response assay against Pf20S (solid lines) and human c20S (dashed lines). Assays were performed in technical triplicate reactions using β5, β2 and β1 specific substrates. **B)** The cryo-EM map density. **C)** Ribbon structure of Pf20S with the β5 subunit highlighted in light blue. In the inset, J-80 (orange) is bound to the β5 catalytic Thr_1_, forming a morpholine ring. Interactions occur between J-80 and the substrate binding pocket of β5 (grey ribbon) and β6 (light blue ribbon).

Previous studies established J-80 as a potent inhibitor of the Pf20S β5 subunit, demonstrating 2,500-fold selectivity in killing *P. falciparum* cultures (IC_50_ = 9.2 ± 1.8 nM) compared to mammalian cell cultures (IC_50_ = 24,300 ± 2,300 nM) [9]. Using our recombinant Pf20S, we confirmed the specificity of J-80 for the β5 subunit (IC_50_ = 22.4 ± 1.3 nM), observing no inactivation of the β2 or β1 subunits at concentrations up to 35 μM (Fig. 5A). Direct comparison of J-80 activity against the human constitutive 20S proteasome (c20S) further revealed that it also targeted the c20S β5 subunit (IC_50_ = 832 ± 63.7 nM) but with 90-fold lower potency than Pf20S β5.

To elucidate the structural basis for J-80’s differential potency against Pf20S β5 versus c20S β5, we determined the structure of J-80 bound to Pf20S by cryo-EM. For this analysis, only correctly assembled mature Pf20S complexes were examined, as J-80 cannot bind to immature complexes lacking a functional active site. Analysis of the cryo-EM data yielded a ~3.0 Å resolution map that shows clear continuous electron density extending from the catalytic threonine of the β5 subunit that resembles J-80. This confirmed that the inhibitor is bound to the β5 subunit (Fig. 5B, **Fig. S5**). Consistent with its biochemical specificity, no substantial electron density was detected in the substrate-binding pockets of the β1 and β2 active sites. Comparison between the apo-Pf20S structure (unbound) and the J-80/Pf20S complex showed good structural alignment, with no global conformational changes induced by J-80 binding (**Fig. S6**).

J-80 is coordinated by multiple residues within the substrate-binding groove at the interface of the β5 and β6 subunits. 1,4-oxazepane forms between the catalytic threonine (Thr1) residue of β5 and the epoxyketone warhead of the inhibitor (Fig. 5C) and this product is stabilized by interactions with Lys33 and Ser130 of the β5 subunit [37]. The inhibitor’s 2,4-difluorobenzene ring at its P1 site occupies a hydrophobic pocket formed by Val31, Met45, and Ala49 of β5, while its P2 mono-fluorobenzene ring coordinates with Cys96 of β5. The P3 *D*-diethyl-asparagine substituent of J-80 is positioned between Met22 of β5 and Val155 of β6, and it also interacts with structural features of the P2 binding pocket. Furthermore, the P4 hexanoyl group of J-80 tucks into a pocket formed by Ser27 and Ser28 of β5, and a β-sheet region of β6 (spanning Asn151 to Cys159). Additionally, the peptide-backbone of J-80 forms multiple hydrogen bonds along the substrate binding groove, interactions that likely stabilize its binding and contribute to its high potency and specificity for Pf20S β5. This structural data further validates the recombinant Pf20S as a suitable platform for therapeutics development.

### Validation of Pf20S-selective inhibitors

**Figure 6:**
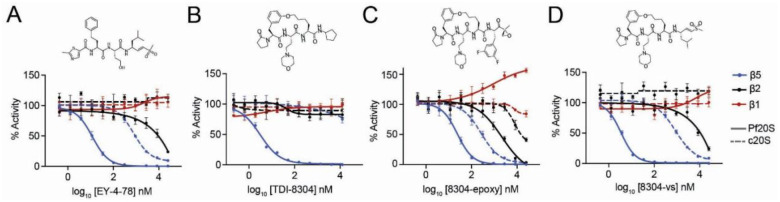
Testing of Pf20S-selective inhibitors. Chemical structure and concentration response assays with **A**. EY-4-78, **B**. TDI-8304, **C**. 8304-EK and **D**. 8304-vs. against Pf20S (solid lines) and human c20S (dashed lines). Assays were performed in technical triplicate reactions using β5, β2 and β1 specific substrates.

Our findings with J-80 demonstrated that targeting the Pf20S β5 subunit was sufficient to kill *P. falciparum*. Consequently, we evaluated additional compounds developed by colleagues at other institutes who were also focused on creating Pf20S-specific proteasome inhibitors. Our initial assessment focused on EY-4-78, a tripeptide-vinyl sulfone inhibitor. This compound was optimized through medicinal chemistry from an earlier generation of Plasmodium-selective tripeptide-vinyl sulfone inhibitors [7] to achieve improved selectivity, solubility, and oral bioavailability [38]. Gel-based competition assays using a fluorescent activity-based probe indicated that EY-4-78 was β5-selective; however, the exact potency of this compound had not yet been determined. In this study, we revealed that EY-4-78 has an IC_50_ of 12.0 ± 0.7 nM for Pf20S β5 and exhibits 65-fold selectivity over c20S β5, although it also targets Pf20S β2 in the micromolar range (Fig. 6A). We also assessed TDI-8304, the lead compound emerging from a medicinal chemistry program aimed at improving the solubility, permeability, and metabolic stability of an initial hit from a series of macrocyclic proteasome inhibitors, while maintaining potency [39]. TDI-8304 was identified in this study as a highly potent and specific inhibitor of Pf20S β5 with IC_50_ = 3.1 ± 0.4 nM (Fig. 6B).

### Structural comparison of TDI

Comparative studies using proteasome mutant lines revealed that TDI-8304 exhibited the highest IC_50_ shifts when compared against J-80 and EY-4-78 [38]. Although these three compounds are structurally distinct, a key differentiating feature is their mode of binding to the β5 subunit: TDI-8304 binds reversibly, whereas J-80 and EY-4-78 bind irreversibly. Furthermore, TDI-8304 demonstrated a higher propensity for inducing resistance *in vitro*. It was postulated that for reversible inhibitors, a mutation-induced reduction in target residence time could be a significant disadvantage, as an irreversible inhibitor might eventually overcome weaker binding affinity to a mutant enzyme if given sufficient time.

When comparing the structures of J-80 and TDI-8304 bound to the β5 subunit of Pf20S there is clear differences between the peptidic and non-peptidic bound ligands. In particular, it was evident that TDI-8304 does not directly interact with the catalytic residue (Thr1) (**Fig. S7**). Based on these observations, two new hybrid inhibitors were developed. These compounds combined the macrocyclic scaffold of TDI-8304 with a P1-warhead group. In this case, either the Leu-vinyl sulfone from EY-4-78 (resulting in 8304-vs) or the 2,4-difluorophenylalanine-epoxyketone from J-80 (resulting in 8304-ek) were synthesized. Studies with these new hybrid compounds revealed that both maintained potent β5 inhibition (IC_50_ = 3.5 ± 0.2 nM for 8304-vs; IC_50_ = 23.7 ± 2.1 nM for 8304-ek), similar to TDI-8304, although they also exhibited some weak inhibition of the β2 subunit in the micromolar range (Fig. 6C–D). Additionally, while the β5 subunit of human c20S was also inhibited, the selectivity for Pf20S β5 over c20S β5 was 33-fold for 8304-vs and 11-fold for 8304-ek. Importantly, these new irreversible-binding hybrid compounds demonstrated a clear reduction in the propensity to acquire resistance *in vitro* [40].

## Discussion:

This study reports the first successful generation of reagent quantities of active, recombinant *P. falciparum* 20S proteasome by co-expressing all 14 subunits with the essential Pf Ump1 chaperone in an insect cell system. This achievement overcomes a long-standing bottleneck in malaria drug discovery caused by the difficulty and low yield of isolating native Pf20S. The availability of a scalable and reliable source of functional Pf20S represents a significant breakthrough for the field.. It opens the door for high-throughput screening of compound libraries, detailed structure-function relationship studies, robust kinetic analyses of inhibitors, and advanced structure-based drug design efforts, all of which were previously challenging. This directly addresses a critical unmet need in the field, similar to how recombinant systems have advanced research on other parasitic enzymes [29–31].

The cryo-EM structural analysis not only validated the correctly assembled mature Pf20S but also provided unprecedented insights into the assembly process since non-catalytic immature complexes were present. This includes the visualization of Ump1 bound to half-Pf20S complexes and the distinct structural conformations of the *Plasmodium* β2 and β5 propeptides compared to their yeast or mammalian counterparts. Furthermore, the unbound β1 C-terminus in the recombinant mature form offers a molecular explanation for the incorrectly assembled proteasomes. These findings shed new light on the intricacies of proteasome biogenesis, highlighting both conserved chaperone dependencies (Ump1) and potentially unique parasite-specific assembly features or vulnerabilities. The tendency for misassembly, even in the presence of Ump1, suggests that *Plasmodium* proteasome assembly might have unique regulatory steps or stability determinants that differ from host or model organisms, which could be areas for future investigation and potential therapeutic targeting.

The extensive biochemical characterization confirms that the recombinant Pf20S, despite containing partially misassembled complexes, recapitulates the properties of the native enzyme. This includes the functionality of all three catalytic subunits (β1, β2, β5) with specific substrates and inhibitors, and appropriate activation by the human PA28α regulator, establishing that the recombinant Pf20S is a reliable and more accessible tool than native isolates for quantitatively assessing inhibitor potency and selectivity. This allows for more standardized and reproducible assays critical for advancing Pf20S-selective inhibitors through the drug development pipeline. The identified optimal substrate panel (Ac-nPnD-amc for β1, Z-VLR-amc for β2, and Ac-FNKL-amc for β5) will be particularly useful for future screening campaigns.

The ~3.0 Å cryo-EM structure of the potent and parasite-selective inhibitor J-80 in complex with recombinant Pf20S provides a detailed molecular understanding of its binding mode and its specificity for the β5 subunit. The structure highlights key interactions within the substrate-binding groove, particularly at the β5/β6 interface, and the formation of a morpholine ring with the catalytic threonine, revealing that the introduction of the *D*-amino acid at the P3 position leads to the change in binding pose, which may contribute the enhanced the selectivity of Pf20S over human proteasomes. These high-resolution structural insights are invaluable for rational drug design. They provide a concrete template for optimizing J-80 or for designing new chemotypes that can achieve even greater potency and selectivity against Pf20S β5 by leveraging these specific interactions, thereby enhancing the therapeutic window for future antimalarial proteasome inhibitors.

The development and characterization of new hybrid inhibitors (8304-vs and 8304-ek), which combine the potent macrocyclic scaffold of a reversible inhibitor (TDI-8304) with irreversible warheads (vinyl sulfone or epoxyketone), represent an innovative chemical strategy. These hybrids successfully retained high potency against Pf20S β5 while demonstrating a marked reduction in the propensity to acquire in vitro resistance compared to their reversible parent compound [40]. This work highlights a promising approach to address the challenge of drug resistance, a major concern in antimalarial therapy. By converting a potent reversible inhibitor into an irreversible one while maintaining target engagement and selectivity, these hybrid compounds offer a pathway to develop more durable antimalarial proteasome inhibitors. This strategy could be more broadly applicable in the development of covalent inhibitors against other parasitic targets where resistance to reversible agents is a concern.

## Materials and Methods:

Bortezomib, carfilzomib, fluorogenic substrates Suc-LLVY-amc, Z-VLR-amc, Z-LLE-amc and Ac-nPnD-amc were purchased from Cayman Chemical. Ac-FNKL-amc was custom-synthesized by GenScript and marizomib was purchased from MedChemExpress. The synthesis of Plasmodium-selective inhibitors is described in the relevant publications [9, 40, 41]. FRSR-ek was synthesized using standard chemistry for peptide epoxyketone inhibitors (**File S3**). Substrates and inhibitors were dissolved in dry DMSO at a concentration of 20 or 10 mM and stored at −20°C. Human constitutive proteasome was purchased from R&D Systems, MN and stored at −80°C.

### Cloning, expression, and protein purification

Complementary DNA sequences encoding 14 proteasome subunits and the assembly chaperone Ump1 from *Plasmodium falciparum* strains Dd2 and DD7 were retrieved from PlasmoDB through sequence alignment against their human orthologs (**Fig. S1**). These sequences underwent codon optimization for *E. coli* expression systems before synthesis by Azenta Life Sciences. The optimized genes were subsequently inserted into three distinct pACEBac1 expression vectors using standard restriction enzyme cloning procedures (**File S2**). Recombinant plasmids were then introduced into DH10EmBacY competent cells (Geneva Biotech) for baculovirus generation. Recombinant protein production was carried out using the baculovirus expression system in Sf9 insect cells (ThermoFisher Scientific, catalog #11496015) following standard protocols. To evaluate the role of the Ump1 chaperone in proteasome assembly, 20S proteasome expression was conducted both with and without co-expression of this protein. Infected Sf9 cells were harvested 72 hours post-infection through centrifugation (2,000 × g, 15 minutes). Cell pellets were resuspended in lysis buffer (Buffer A: 50 mM Tris-HCl pH 7.5, 150 mM NaCl, 1 mM dithiothreitol (DTT), 1 mM EDTA) at a 4:1 volume ratio. Cell disruption was achieved through sonication, followed by clarification via high-speed centrifugation (20,000 × g, 20 minutes). The clarified cell extract was applied to tandem Streptactin XP affinity columns (QIAGEN) pre-equilibrated with Buffer A. After thorough washing, bound proteins were eluted using Buffer A containing 50 mM biotin. Proteasome-enriched fractions were pooled and concentrated via ultrafiltration (Amicon, 100 kDa) before size exclusion chromatography on a Superose 6 Increase 10/300 column (GE Healthcare). The column was equilibrated with gel filtration buffer (50 mM HEPES pH 7.5, 150 mM NaCl, 1 mM EDTA), and 0.5 ml fractions were collected for protease activity analysis. Enzymatically active fractions were combined and concentrated for further analysis.

### Proteasome activity and inhibition assays

Catalytic activity of individual proteasome subunits was assessed using subunit-selective fluorogenic peptide substrates. For human c20S proteasome, the β1, β2, and β5 active sites were monitored using Z-LLE-amc, Z-VLR-amc, and Suc-LLVY-amc substrates, respectively. The corresponding *Plasmodium falciparum* Pf20S subunits were evaluated with Ac-nPnD-amc, Z-VLR-amc, and Ac-FNKL-amc substrates. Enzymatic assays were performed in 384-well microplate format. Human c20S proteasome (1 nM) was pre-activated with 100 nM PA28α human activator, while Pf20S (1 nM) required 200 nM PA28α activator for optimal activity. The PA28α activator protein was produced recombinantly in *E. coli* using pSumo expression vector following established protocols [42]. For c20S assays, the reaction mixture comprised of 50 mM HEPES buffer (pH 7.5) and 1 mM DTT, with fluorogenic substrates at the following concentrations: 80 μM Z-LLE-amc, 30 μM Z-VLR-amc, and 65 μM Suc-LLVY-amc. Each reaction well contained 8 μL total volume. Pf20S enzymatic reactions utilized a modified buffer system containing 50 mM HEPES (pH 7.5), 5 mM MgCl_2_, 1 mM DTT and 0.01% BSA. Substrate concentrations were optimized to 80 μM Ac-nPnD-amc, 25 μM Z-VLR-amc, and 50 μM Ac-FNKL-amc, maintaining 8 μL reaction volumes. Test compounds were precisely dispensed using an Echo650 acoustic liquid handler (Beckman Coulter). Kinetic analysis protocols were tailored to inhibitor mechanism: irreversible inhibitors underwent pre-steady-state measurements with IC_50_ determination at 60–90 minutes post-reaction initiation, while reversible inhibitors were subjected to steady-state kinetics following 30-minute pre-incubation periods. All experiments were conducted in triplicate using black 384-well microplates (Greiner Bio-One, Monroe, NC) maintained at 37°C. Fluorescence detection employed a Synergy HTX Multi-Mode Microplate Reader (BioTek, Winooski, VT) with excitation/emission wavelengths set to 360/460 nm. Data processing and statistical analysis were performed using GraphPad Prism software (version 10.4.1).

### Protein gels and active site probing

Recombinant Pf20S and Pf20S-inhibitor complex were with 2 μM Me4BodipyFL-Ahx3Leu3VS (R&D Systems #I-190). After probe addition, samples were incubated at room temperature for 16 h. For denaturing gels, samples were mixed with 4X Bolt LDS sample buffer (ThermoFisher Scientific) containing 250 μM DTT, heated at 99°C for 5 min, and loaded onto a NuPAGE 12% Bis-Tris gel (ThermoFisher Scientific). PageRuler Plus pre-stained protein ladder (ThermoFisher Scientific) was included on each gel. Gels were run with 1X MOPS SDS buffers (Invitrogen) at 120 V. For native gels, samples were mixed with 2X Novex Tris-glycine native sample buffer and loaded onto NuPAGE 3–8% Tris-glycine gels (Invitrogen) with NativeMark unstained protein standard (Thermo Fisher Scientific, 57030). Gels were run at 100 V with Novex Tris-glycine running buffer (Invitrogen). All gels were imaged on Bio-Rad ChemiDoc XRS+ at 470 nm excitation and 530 nm emission for Me4BodipyFL-Ahx3Leu3VS probe visualization and silver stained. Bands were quantified using Image Lab 6.1.

### In gel digestion and desalting

Bands for proteomic analysis were excised from a native gel and processed according to [43]. The extracted digest was dried and resuspended in 0.1% formic acid before C18 ZipTip desalting. A C18 column was washed with methanol and spun for 45 s at 3500 × g. The column was equilibrated with 0.1% formic acid (FA) in 50% acetonitrile followed by 0.1% FA in water. The sample was loaded onto the column and centrifuged for 2 min at 2000 × g. It was then washed with 0.1% FA then eluted from C18 with 50% acetonitrile, 0.1% FA by spinning at 3500 x g for 45 s. The sample was dried in a Savant Speed Vac Plus AR and stored at −80°C.

### LC/MS Analysis

LC separation was performed using a Dionex Ultimate 3000 nano HPLC system (Thermo Scientific) online coupled to the mass spectrometer. Samples were loaded onto a trap column (C18 PepMap100, 5 μm particle size, 300 μm × 5 mm, ThermoFisher Scientific) for 2 min at a flow rate of 25 μL/min. The loading buffer consisted of water with 2% acetonitrile and 0.1% trifluoroacetic acid. Peptides were separated using a linear gradient of mobile phase B from 5% to 50% over 17 min. Mobile phase A consisted of water with 0.1% FA, and mobile phase B consisted of acetonitrile with 0.1% FA. Chromatographic separation was achieved using a nano reversed-phase column (Aurora Ultimate TS, 25 cm × 75 μm ID, 1.7 μm particle size, Ion Opticks). Mass spectrometric analysis was performed on a Thermo Scientific Orbitrap Fusion mass spectrometer operating in data-dependent acquisition (DDA) mode. Eluting peptide cations were ionized by electrospray ionization in positive mode with a spray voltage of 1600 V and ion transfer tube temperature of 275°C. MS1 survey scans were acquired in the Orbitrap analyzer over the mass range of 350–1400 m/z at 120,000 resolution with the following parameters: RF lens 60%, maximum injection time 246 ms, and AGC target 1 × 10^5^. Precursor ions with charge states 2–7 and intensity >5000 were selected for fragmentation. Dynamic exclusion was applied for 5 s with a mass tolerance of 10 ppm. Selected precursor ions were isolated using the quadrupole with a 1.6 m/z isolation window and fragmented by higher-energy collisional dissociation (HCD) with 30% normalized collision energy. Fragment ions were detected in the ion trap with an AGC target of 1 × 10^4^ and maximum injection time of 35 ms. Raw MS data were processed using Thermo Proteome Discoverer software (version 3.2.0.450) with the Sequest HT search algorithm. Peptide identification was performed against the combined *Plasmodium falciparum* and *Spodoptera frugiperda* proteome database (UniProt proteome IDs: UP000001450 and UP000829999, respectively) supplemented with common contaminants. Search parameters included carbamidomethylation of cysteine as a fixed modification and methionine oxidation as a variable modification. Trypsin was specified as the digestion enzyme with a maximum of 2 missed cleavages allowed. Precursor mass tolerance was set to 10 ppm and fragment mass tolerance to 0.6 Da. Protein and peptide false discovery rates (FDR) were controlled at 1% using the Percolator algorithm.

### Preparation of cryo-EM grids and data acquisition

Pf20S with J-80 inhibitor was prepared in 50 mM HEPES pH 7.5, 1 mM TCEP, and 260 μM J-80. The solution was incubated at room temperature for 1 h. Phosphocholine was added to a final concentration of 0.25 mM prior to cryo-EM grid preparation. Quantifoil Cu 300 mesh R2/1 grids were glow-discharged using a Pelco easiGlow system at 15 mA for 25 seconds to enhance surface hydrophilicity. 2.5 μL aliquot of the purified Pf20S or Pf20S:J-80 complex was applied to the grid, blotted for 8 seconds with a blot force of 0, using Whatman 1 filter paper at 4 °C and 100% humidity, and then plunge-frozen in liquid ethane using a Vitrobot Mark IV (ThermoFisher Scientific). Vitrified samples were imaged in a Titan Krios electron microscope (FEI/ThermoFisher) in the Cryo-EM facility of the Structural Biology Shared Resource at Sanford Burnham Prebys Medical Discovery Institute. Images were collected on a Gatan K3 detector using SerialEM [44] under super-resolution settings [45].

### Cryo-EM image processing

A total of 3,560 raw cryo-EM images were collected for the apo Pf20S sample, and 9,270 raw images were collected for Pf20S complexed with J-80. Image processing was performed separately for the apo and J-80–bound datasets using cryoSPARC (v4.7.0) [46, 47]. Motion correction of the movies was carried out using the Patch Motion Correction tool with output F-crop factor 1/2. CTF estimation was performed using the Patch CTF tool with minimum search defocus of 10,000 Å, and maximum search defocus of 30,000 Å, respectively. Initial particle picking was conducted using the Blob Picker tool, with a minimum particle diameter of 100 Å, a maximum diameter of 150 Å. The particles were extracted with a box size of 400 Å and Fourier crop to box size 100 Å and subjected to 2D classification into 50 classes. High-quality 2D classes were selected and used as templates for further particle picking. Multiple rounds of 2D classification were carried out to remove unwanted particles and enrich for well-aligned particle images. High-quality side-view 2D classes of full Pf20S were selected and re-extracted with a box size of 400 Å. Non-uniform Refinement was carried out to generate the final 3D maps.

Image processing of the half-Pf20S was performed using the Pf20S: J-80 dataset due to the larger size of this dataset compared to the apo Pf20S dataset. High-quality 2D classes of tilted half-Pf20S were selected, subjected to additional rounds of 2D classification, and re-extracted using a smaller box size of 200 Å. These particles were subsequently used to generate 3D maps using the same workflow as above.

### Model building and refinement

A high-resolution Pf20S structure (PDB: 7LXU) was used as an initial template to build the apo Pf20S model. The initial atomic model was fitted into the cryo-EM density map using the ‘Fit in Map’ tool in UCSF Chimera [48]. Real-space refinement was performed residue by residue in Coot using the “real space refine zone” tool [49, 50]. The resulting model was further refined in Phenix using real-space refinement with the following parameters: maximum iterations set to 100, five macro-cycles, target bond RMSD of 0.01 Å, target angle RMSD of 1.0°, and with secondary structure restraints enabled. Model validation was conducted using the comprehensive validation tools in Phenix [51, 52]. The refinement process involved iterative rounds between Coot and Phenix to achieve satisfactory validation metrics.

To facilitate docking, J-80 was combined with the side chain of Thr_1_ from the β5 subunit as a single covalently linked structure. The J-80–Thr_1_ adduct was constructed using the Ligand Builder tool in Coot, and its corresponding geometric restraints CIF file was generated using eLBOW in Phenix with the “simple optimization” option. The J-80–Thr_1_ model was docked into the apo Pf20S structure using UCSF Chimera by aligning the Thr1 from J-80-Thr with the Thr1 of β5 subunit from the apo Pf20S. The original β5 Thr1 residue was deleted from the apo structure, and the merged J-80–Thr1 and Pf20S model was used to create the initial J-80–Pf20S model. A local refinement around the ligand binding site was performed in Coot to optimize the fit of J-80 within the cryo-EM density. Final refinement was carried out in Coot and Phenix as described above. The apo Pf20S model served as the initial template to build the half-Pf20S structure. Fitting and refinement of this tilted half-Pf20S model were carried out in Coot and Phenix as described above. All final cryo-EM maps and models were visualized using UCSF Chimera and ChimeraX [53].
